# Optimal Power Control in Wireless Powered Sensor Networks: A Dynamic Game-Based Approach

**DOI:** 10.3390/s17030547

**Published:** 2017-03-09

**Authors:** Haitao Xu, Chao Guo, Long Zhang

**Affiliations:** 1 School of Computer and Communication Engineering, University of Science and Technology Beijing; Beijing 100083, China; 2 Communication Engineering Department, Beijing Electronics Science and Technology Institute, Beijing 100070, China; guo99chao@163.com; 3 School of Information and Electrical Engineering, Hebei University of Engineering, Handan 056038, China; zhanglong@hebeu.edu.cn

**Keywords:** differential game, power control, WPSN

## Abstract

In wireless powered sensor networks (WPSN), it is essential to research uplink transmit power control in order to achieve throughput performance balancing and energy scheduling. Each sensor should have an optimal transmit power level for revenue maximization. In this paper, we discuss a dynamic game-based algorithm for optimal power control in WPSN. The main idea is to use the non-cooperative differential game to control the uplink transmit power of wireless sensors in WPSN, to extend their working hours and to meet QoS (Quality of Services) requirements. Subsequently, the Nash equilibrium solutions are obtained through Bellman dynamic programming. At the same time, an uplink power control algorithm is proposed in a distributed manner. Through numerical simulations, we demonstrate that our algorithm can obtain optimal power control and reach convergence for an infinite horizon.

## 1. Introduction

Conventional wireless sensor networks (WSN) are always disposable systems, because sensors cannot be recharged due to random deployment and the entire network is invalid when the batteries of wireless sensors run out of energy [1]. As the use of the WSN is strictly limited by the life span of the sensors’ batteries, energy consumption has become one of the biggest constraints of the wireless sensor node and has posed many challenges to WSNs [2]. Energy has become one of the scarcest resources in WSN [3]. Wireless power transfer (WPT) and other energy harvesting technologies provide solutions in such situations. Benefiting from microwave wireless power transfer, the wireless powered sensor networks (WPSN) can be used to reduce the operational cost, provide a stable energy supply, and achieve much longer operating lifetimes [4]. 

WPSN has been widely researched in the recent literature [5,6,7,8]. Wireless sensors in WPSN can be powered through WPT in the downlink which is radio frequency enabled, and can use the harvesting energy for information transmission in the uplink. Compared to other energy harvesting technologies, WPT can achieve long-distance energy transfer and constant energy supplementation [6]. However, in WPSN, the distance between the wireless sensors and energy nodes (ENs) may cause performance unfairness, because of the near-far effect. When wireless sensors are located far away from the ENs, they will receive less energy, because of power transmission attenuation. But they may need more energy for the uplink information transmission. Thus, communication and energy scheduling should be considered on this occasion. For the downlink energy transfer, energy beamforming technology is used for sensors which are located far away from energy sources, so that they can receive stronger energy beams [7]. In this case, it is essential to research the uplink transmit power control problem, to balance throughput and energy performance among different sensors [8]. 

When considering the literature, many studies have been completed in WPSN for power control problems. In [9], the information relay nodes were working as energy beacon and information relays, and that energy and information can be transferred and transmitted through relay nodes. The protocol was divided into three phases. By jointly optimizing the duration and power allocation for transmission, the network throughput was maximized. In [10], the authors researched the energy harvesting-based wireless networks and proposed an iterative method-based solution for the sub-channel and power allocation. The authors defined a logarithmic utility function, considering both the aggregated rate and the harvested energy. The sub-channel and power allocation are obtained through biconvex optimization. The convergence of the proposed algorithm is also proved. 

Game theory solves the resource allocation problem of a system with conflicting components. It has recently received an increasing interest in the context of wireless sensor networks [11,12]. It can be used to solve the optimal energy management problems in wireless powered sensor networks, to meet the perceived QoS (Quality of Services) performance [13]. In [13], the wireless energy request policy was researched and analyzed, based on a constrained stochastic game model. A constrained Nash equilibrium solution was obtained, while meeting Qos requirements, and achieved an energy request cost minimization. In [14], in WPSN, a Nash bargaining-based optimal power control approach was proposed, to balance the information transmission efficiency. The whole game process was simplified into three parts, and the power control and time allocation algorithm were proved to be quasiconcave.

Nevertheless, to the best of our best knowledge, all of the works above optimize the network performance, but do not consider the dynamic characteristics of a sensor’s energy which are exponent variables, and do not consider the optimization in given a time period. Differential Game, firstly proposed by Isaace [15], is one of the most practical and complex branches of game theory and can be used to solve a class of resource allocation problems, under which the evolution of the state is described by a differential equation and the players act throughout a time interval [16]. In this paper, we propose a new method for uplink transmit power control, based on a differential game. Each sensor node can be satisfied with a constant wireless energy transfer from a hybrid access point, and the hybrid access point has an energy transfer function and information transmission function. The energy dynamic of sensors is considered as the state of the system, which is denoted by differential equations. We suppose that all sensors are rational players and the combination of energy and throughput revenues are interpreted as the optimization objectives for different players. We will obtain individual feedback Nash equilibriums for the sensors in a finite time horizon and those in an infinite time horizon, and an iterative algorithm is presented to achieve optimal solutions for uplink power control. The numerical results will be given, to present the correctness of the differential game analysis.

The remainder of the paper is organized as follows. Section 2 introduces the system model of WPSN and the uplink power control problem in a differential game. Section 3 provides feedback Nash equilibrium solutions for each wireless sensor and a differential game-based iterative algorithm. Numerical simulations are given in Section 4. Finally, we conclude the work in Section 5. 

## 2. System Model and Problem Formulation

**Figure 1:**
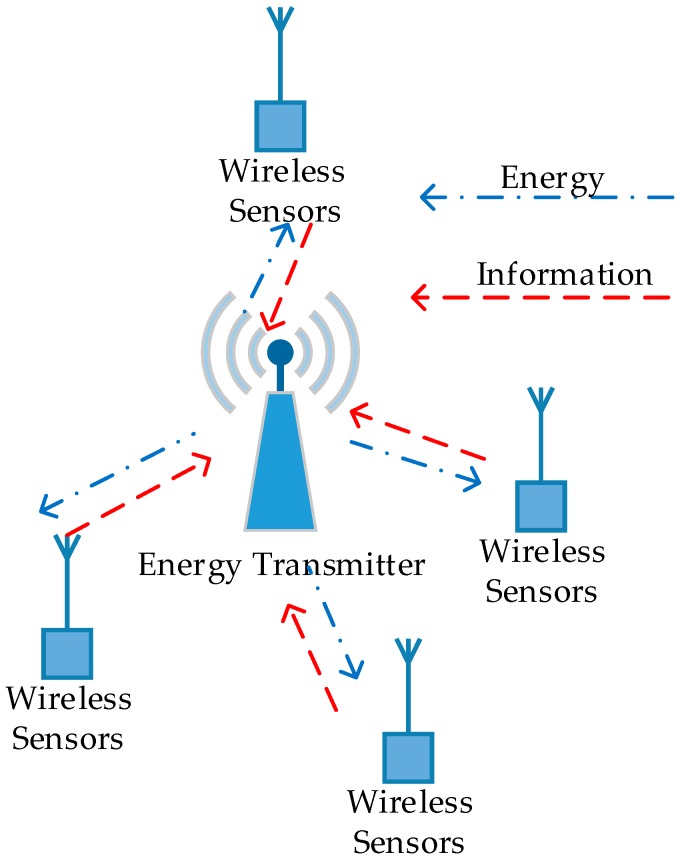
System model for a WPSN.

In this section, we propose a differential game model for uplink transmit power control in wireless powered sensor networks (WPSN). We consider a WPSN where there is one wireless energy transmitter serving several wireless sensors in its coverage area (as shown in Figure 1). The wireless energy transmitter can achieve a constant power supply in the downlink and can work as an access point for information transmission in the uplink, receiving signals from distributed wireless sensors. Thus, the wireless energy transmitter can be considered as a hybrid access point (H-AP) for energy and information transmission. All of the devices (including wireless energy transmitters and wireless sensors) in WPSN are assumed to work on orthogonal frequency bands, and work in the half-duplex mode. The wireless sensors use the energy harvested from H-AP for information transmission [17]. The energy harvested by each sensor is stored in a rechargeable battery and then used for wireless information transmission (WIT). Moreover, the wireless sensors control their uplink transmit power to extend the working hours, meanwhile improving their own QoS, which is a distributed optimization problem and leads to a dynamic game that can be modeled by a non-cooperative differential game.

**Figure 2:**
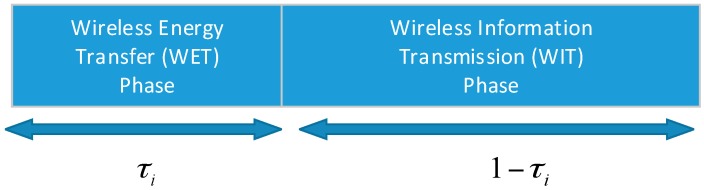
Two-step transmission phase.

In this paper, the “harvest-then-transmit” protocol [18] is considered. The time duration of transmission is assumed to be a different block transmission time with a normalized duration. Energy and information are transmitted from block to block. For each transmission block, it can be divided into two phases (as shown in Figure 2). The first phase is the time duration of wireless energy transfer (WET), which is denoted by $\tau_{i}$, and the second phase is the time duration of wireless information transmission (WIT), which is represented as $1-\tau_{i}$. 

Our target is to control the uplink transmit power of sensors in WPSN, to maximize the sensors’ own economic revenue during the time period t∈[0,T]. A differential game-based model is constructed to describe the revenue maximization problem. Through the optimal power control, the wireless sensors in WPSN can achieve a balance between energy consumption and QoS improvement. In order to simplify the system, we will consider a WPSN with one H-AP and N wireless sensors, where N is the set of wireless sensors (players). During the first phase of wireless energy transfer, let $p_{T}^{i}$ denote the transfer power from H-AP to sensor i. It is assumed that $p_{T}^{i}$ satisfies a maximum power constraint $P_{T}^{\max}$ (i.e., $0\leq p_{T}^{i}\leq P_{T}^{\max}$) [19]. The harvesting energy in sensor i is given by [20]:

(1) $$E_{h}=\eta_{i}\tau_{i}{\left\|g_{T}^{i}\right\|}^{2}p_{T}^{i},\;x_{i}\left(0\right)=0$$

where $\eta_{i}$ is the energy conversion efficiency of player i, and $0<\eta_{i}\leq 1$. ${\left\|g_{T}^{i}\right\|}^{2}$ is the downlink channel gain. Let $x_{i}\left(t\right)$ denote the power level of player i, which can be interpreted as the state variables of a system. State variables are dynamic variables over different time periods that are influenced by the uplink transmit power, as well as by exiting levels of the state variables. Let $p_{i}\left(t\right)$ denote the uplink transmit power of player i, which is viewed as the control variable. The dynamic of the power level can be characterized as a linear differential equation, i.e.:

(2) $$dx_{i}=\left[-\mu_{i}x_{i}-\left(1-\tau_{i}\right)p_{i}+\eta_{i}\tau_{i}{\left\|g_{T}^{i}\right\|}^{2}p_{T}^{i}\right]dt,\;x_{i}\left(0\right)=0$$

where $\mu_{i}$ is the energy loss coefficient. $x_{i}\left(0\right)=0$, is the initial state, which means that there is no energy transmission at the beginning of the game. 

Now, we discuss how wireless sensors control their uplink transmit power to achieve revenue maximization, to reach an equilibrium between energy consumption and an achievable throughput. Subject to the limited energy, each sensor aims to minimize the uplink transmit power to extend the working hours, but may result in less information transmission and a low QoS. Therefore, each sensor needs to balance the conflict between energy consumption and an achievable throughput. Generally speaking, there will be a queue length or buffer size for each H-AP. When the buffer size of the H-AP is full, it will refuse to provide the service for any uplink information transmission. Therefore, in our game, we suppose that there are enough buffer sizes for information transmission and only consider how to control the uplink transmission power to achieve revenue maximization. The structure of the optimization model will consist of energy revenue specifications and QoS revenue specifications. 

Firstly, we give the energy revenue definition. The energy revenue depends on the energy storage in the sensors and the energy’s unit price. Assuming the unit price is ε, the instantaneous energy revenue is defined as a linear form, as follows:

(3) $$U_{i}^{eng}=\varepsilon x_{i}$$

In perfect competition, each sensor will use the lowest power possible, to reduce the energy consumption and increase the energy revenue, given by Equation (2). However, less transmission power may cause a low transmission rate and low QoS. Thus, we introduce a QoS revenue to describe the conflict between energy consumption and QoS requirements. As the “harvest-then-transmit” protocol is considered, there is no interference from the energy transmission. Let the achieve rate of sensor i denote the QoS revenue, where the QoS revenue specifications are obtained as:

(4) $$U_{i}^{QoS}=\rho \left(1-\tau_{i}\right)\log\left(1+\frac{g_{i}p_{i}}{\sigma_{i}^{2}}\right)=\rho \left(1-\tau_{i}\right)\log\left(1+\gamma_{i}p_{i}\right)$$

where $\gamma_{i}=g_{i}/\sigma_{i}^{2}$, $p_{i}$ is the uplink transmit power and the control variables of the game. $g_{i}$ is the uplink channel power gain. ρ is a constant parameter that denotes the unit rate revenue. 

Based on the above assumption, the total revenue of wireless sensor i is denoted as follows:

(5) UiRevenue=UiQoS+UiEng=ρ(1−τi)log(1+γipi)+εxi

In this paper, we use the noncooperative differential game theory [21] to analyze the optimal uplink transmit power and to achieve revenue maximization for each sensor. Let the target QoS level for each sensor be denoted by $S_{i}$. We evaluate the balance between energy consumption and QoS over the time interval [0,T], using the term $\alpha_{i}\left(x_{i}\left(T\right)-S_{i}\right)$, where $\alpha_{i}$ is a constant parameter and T is the end of the control. Let r denote the discount rate, where the dynamic game of the power control for each sensor noncooperatively chooses its uplink transmit power as:

(6) $$\begin{array}{ll} L_{i} & =\underset{p_{i}}{\max}\int_{0}^{T}U_{i}^{\mathrm{Pr}ofit}\left(t\right)e^{-rt}dt+U_{i}^{Termal}\left(t\right)\\ & =\underset{p_{i}}{\max}\int_{0}^{T}\left[\rho \left(1-\tau_{i}\right)\log\left(1+\gamma_{i}p_{i}\right)+\varepsilon x_{i}\right]e^{-rt}dt+\alpha_{i}\left(x_{i}\left(T\right)-S_{i}\right) \end{array}$$

Subject to the deterministic dynamics:

(7) $$dx_{i}=\left[-\mu_{i}x_{i}-\left(1-\tau_{i}\right)p_{i}+\eta_{i}\tau_{i}{\left\|g_{T}^{i}\right\|}^{2}p_{T}^{i}\right]dt$$

Now, we formulate the optimal power control for all sensors in WPSN as a differential game, as follows.

- Players : All wireless sensors i∈N in the WPSN.
- Strategy space: All wireless sensors can noncooperatively choose their uplink transmit power $\left\{p_{i}^{*}\left(t\right)\right\}$, to maximize the revenue.
- State: The power level state is denoted by vector $x_{i}\left(t\right)$, where the state is controlled by the dynamic constraint in Equation (2).
- Objective function: All of the wireless sensors act to maximize their discounted revenues over a time interval [0,T], respectively.

## 3. Game Analysis

In this section, we analyse the optimal uplink transmit power for each wireless sensor. In the following subsections, we first discuss the optimal uplink transmit power in a finite horizon. Then, the optimal strategy will be considered under an infinite horizon. An uplink power control algorithm based on the differential game will be given in the third subsection.

### 3.1. Analysis of Differential Game in Finite-Horizon 

The finite horizon differential game will be solved, based on the dynamic optimization program technique, which was developed by Bellman [22,23]. According to Bellman’s dynamic programming principle, the uplink transmit power should be optimal for the given time duration.

**Lemma** **1.** *For the optimization Equations (6) and (7), an n-tuple of strategies* $\left\{p_{i}^{*}\left(t,x\right),for\;i\in N\right\}$ *constitutes a feedback Nash equilibrium solution if there exists a functional* $V^{i}\left(t,x\right)$*, defined on the time interval* [0,T] *and satisfying the following relations for each* i∈N [22,23]:

(8) $$\begin{array}{l} V^{i}\left(t,x\right)=\int_{t}^{T}\left[\rho \left(1-\tau_{i}\right)\log\left(1+\gamma_{i}{p_{i}}^{*}\right)+\varepsilon {x_{i}}^{*}\right]e^{-rs}ds+\alpha_{i}\left(x_{i}^{*}\left(T\right)-S_{i}\right)\\ \geq \int_{t}^{T}\left[\rho \left(1-\tau_{i}\right)\log\left(1+\gamma_{i}p_{i}\right)+\varepsilon x_{i}\right]e^{-rs}ds+\alpha_{i}\left(x_{i}\left(T\right)-S_{i}\right) \end{array}$$

(9) $$V^{i}\left(T,x\right)=\alpha_{i}\left(x_{i}\left(T\right)-S_{i}\right)$$

*where the time interval* [0,T]*:*

(10) $$dx_{i}^{*}=\left[-\mu_{i}x_{i}^{*}-\left(1-\tau_{i}\right)p_{i}^{*}+\eta_{i}\tau_{i}{\left\|g_{T}^{i}\right\|}^{2}p_{T}^{i}\right]dt$$

For all t∈[0,T], if the strategies $\left\{p_{i}^{*}\left(s\right),for\;i\in N\right\}$ provide a feedback Nash equilibrium to the differential game problem on the time interval [0,T], it can provide a feedback Nash equilibrium for the same problem on the time interval [t,T].

**Lemma** **2.** *A feedback Nash equilibrium solution to the games (6) and (7) has to satisfy the following conditions:*

(11) $$\begin{array}{l} -V_{t}^{i}\left(t,x\right)=\underset{p_{i}}{\max}\{\left[\rho \left(1-\tau_{i}\right)\log\left(1+\gamma_{i}{p_{i}}^{*}\right)+\varepsilon {x_{i}}^{*}\right]e^{-rt}\\ +V_{x}^{i}\left(t,x\right)\left(-\mu_{i}x_{i}-\left(1-\tau_{i}\right)p_{i}^{*}+\eta_{i}\tau_{i}{\left\|g_{T}^{i}\right\|}^{2}p_{T}^{i}\right)\} \end{array}$$

(12) $$V^{i}\left(T,x\right)=e^{-rT}\alpha_{i}\left(x_{i}\left(T\right)-S_{i}\right)$$

**Lemma** **3.** *In the wireless information phase, the optimal uplink transmit power for each sensor* i∈N *in WPSN, satisfies:*

(13) $$p_{i}^{*}\left(s\right)=\rho \left[\left(\alpha_{i}-\frac{\varepsilon }{r+\mu_{i}}\right)e^{\left(r+\mu_{i}\right)\left(t-T\right)}-\frac{\varepsilon }{r+\mu_{i}}\right]-\frac{1}{\gamma_{i}}$$

**Proof.**  See Appendix A. ☐

### 3.2. Analysis of Infinite-Horizon Differential Game

Consider the infinite-horizon autonomous game problem with constant discounting, in which T approaches infinity and where the objective functions and state dynamics are both autonomous. Now consider the alternative game to (6) and (7):

(14) $$L_{i}=\underset{p_{i}}{\max}\int_{0}^{\infty }U_{i}^{\mathrm{Pr}ofit}\left(t\right)e^{-rt}dt=\underset{p_{i}}{\max}\int_{0}^{\infty }\left[\rho \left(1-\tau_{i}\right)\log\left(1+\gamma_{i}p_{i}\right)+\varepsilon x_{i}\right]e^{-rt}dt$$

Subject to the deterministic dynamics:

(15) $$dx_{i}=\left[-\mu_{i}x_{i}-\left(1-\tau_{i}\right)p_{i}+\eta_{i}\tau_{i}{\left\|g_{T}^{i}\right\|}^{2}p_{T}^{i}\right]dt,\;x_{i}\left(0\right)=0$$

The infinite-horizon autonomous game is independent of the choice of t and only dependent upon the state at the starting time, which is 0. Then, a feedback Nash equilibrium solution for the infinite-horizon autonomous games (14) and (15) can be characterized as follows:

**Lemma** **4.** *An n-tuple of strategies* $\left\{q_{i}^{*}\left(x\right),for\;i\in N\right\}$ *constitutes a feedback Nash equilibrium solution if there exists a functional* $W^{i}\left(x\right)$ *, defined on the time interval* [0,T] *and satisfying the following set of partial differential equations for each* i∈N*:*

(16) $$rW^{i}\left(x\right)=\underset{p_{i}}{\max}\left\{\rho \left(1-\tau_{i}\right)\log\left(1+\gamma_{i}q_{i}\right)+\varepsilon x_{i}+W_{x}^{i}\left(x\right)\left(-\mu_{i}x_{i}-\left(1-\tau_{i}\right)q_{i}+\eta_{i}\tau_{i}{\left\|g_{T}^{i}\right\|}^{2}p_{T}^{i}\right)\right\}$$

**Lemma** **5.** The optimal uplink power for each wireless sensor is independent of the time, which is the game equilibrium strategy and can be expressed as:

(17) $${q_{i}}^{*}=\frac{\rho }{\varepsilon }\left(r+\mu_{i}\right)-\frac{1}{\gamma_{i}}$$

**Proof.**  See Appendix B. ☐

**Lemma** **6.** The optimal strategy for the infinite-horizon differential game satisfies:

(18) $$\begin{array}{l} x_{i}=\left(\frac{\rho \left(1-\tau_{i}\right)\left(r+\mu_{i}\right)}{\mu_{i}\varepsilon }-\frac{\left(1-\tau_{i}\right)}{\mu_{i}\gamma_{i}}-\frac{\eta_{i}\tau_{i}{\left\|g_{T}^{i}\right\|}^{2}p_{T}^{i}}{\mu_{i}}\right)e^{-\mu_{i}t}\\ -\frac{\rho \left(1-\tau_{i}\right)\left(r+\mu_{i}\right)}{\mu_{i}\varepsilon }+\frac{\left(1-\tau_{i}\right)}{\mu_{i}\gamma_{i}}+\frac{\eta_{i}\tau_{i}{\left\|g_{T}^{i}\right\|}^{2}p_{T}^{i}}{\mu_{i}} \end{array}$$

**Proof.**  Substituting the optimal uplink power obtained in Equation (17), which is also the game equilibrium strategy, into the state function, yields:

(19) $$dx_{i}=\left[-\mu_{i}x_{i}-\left(1-\tau_{i}\right)\left(\frac{\rho }{\varepsilon }\left(r+\mu_{i}\right)-\frac{1}{\gamma_{i}}\right)+\eta_{i}\tau_{i}{\left\|g_{T}^{i}\right\|}^{2}p_{T}^{i}\right]dt,\;x_{i}\left(0\right)=0$$

☐

The optimal state trajectory can be obtained through solving the above dynamics, and is denoted as:

(20) $$\begin{array}{l} x_{i}=\left(\frac{\rho \left(1-\tau_{i}\right)\left(r+\mu_{i}\right)}{\mu_{i}\varepsilon }-\frac{\left(1-\tau_{i}\right)}{\mu_{i}\gamma_{i}}-\frac{\eta_{i}\tau_{i}{\left\|g_{T}^{i}\right\|}^{2}p_{T}^{i}}{\mu_{i}}\right)e^{-\mu_{i}t}\\ -\frac{\rho \left(1-\tau_{i}\right)\left(r+\mu_{i}\right)}{\mu_{i}\varepsilon }+\frac{\left(1-\tau_{i}\right)}{\mu_{i}\gamma_{i}}+\frac{\eta_{i}\tau_{i}{\left\|g_{T}^{i}\right\|}^{2}p_{T}^{i}}{\mu_{i}} \end{array}$$

### 3.3. Uplink Power Control Algorithm

In this subsection, we present an uplink power control algorithm (Algorithm 1) in wireless powered sensor networks, based on the infinite-horizon solutions presented in Section 3.2, which is as follows:

**Algorithm 1. The strategy for each sensor to determine the optimal uplink transmit power**1:Initially, sensor set the power level x as 0, there is no energy transmission at the beginning of the game.2:**for** sensor i∈N
**do**3: Start game, initial parameters $\tau_{i}$,$\mu_{i}$,$\eta_{i}$ for the game;4: Based on the QoS requirements, set the final rate revenue level as $S_{i}$
5: **while**
$x_{i}>0$, **do**6: Calculate the optimal uplink power based on Equation (17);7: Calculate the optimal strategy of power level based on Equation (20);8: Calculate the maximized revenue for each sensor based on Equations (14), (17) and (20);9: Updata power level $x_{i}$ for each sensor;10: **end while**11:**end for**

In the above algorithm, each sensor continues to calculate the optimal uplink transmit power, until there is no energy left in the sensor’s batteries for information transmission. 

## 4. Numerical Simulations

### 4.1. Optimal Power and Revenue 

**Figure 3:**
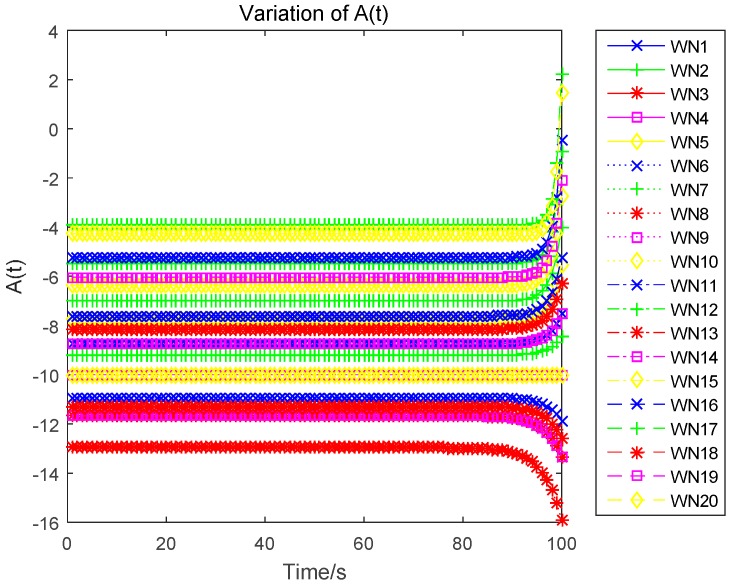
Variation of $A_{i}\left(t\right)$ with time.

**Figure 4:**
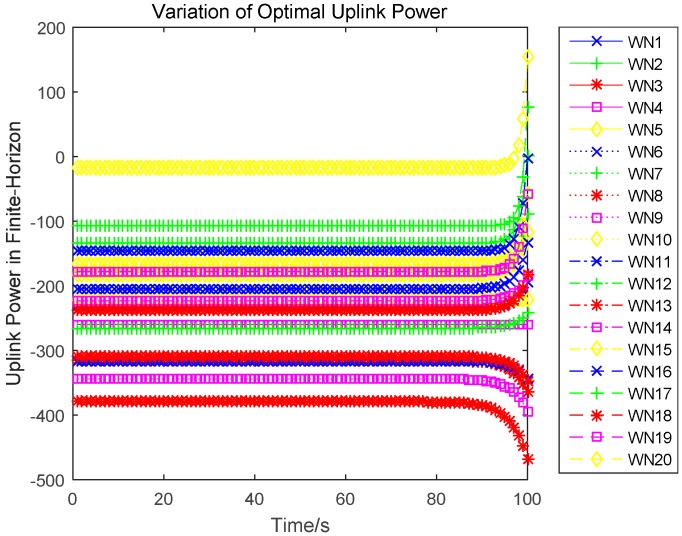
Variation of the optimal uplink power in finite-horizon.

**Figure 5:**
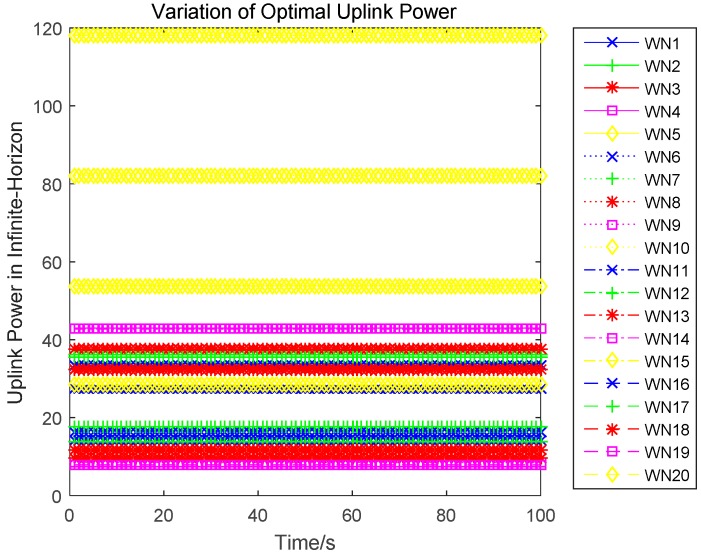
Variation of the optimal uplink power in infinite-horizon.

**Figure 6:**
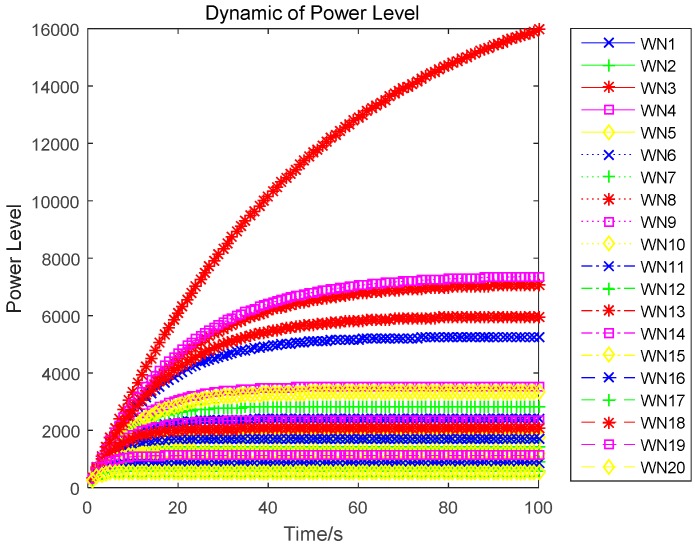
Dynamic of power level (the optimal state trajectory).

**Figure 7:**
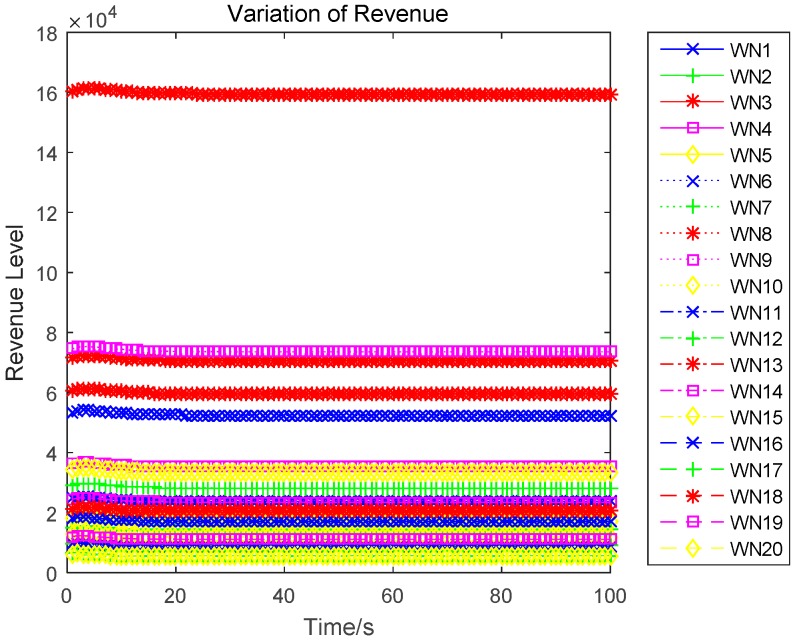
Dynamic of revenue level.

**Figure 8:**
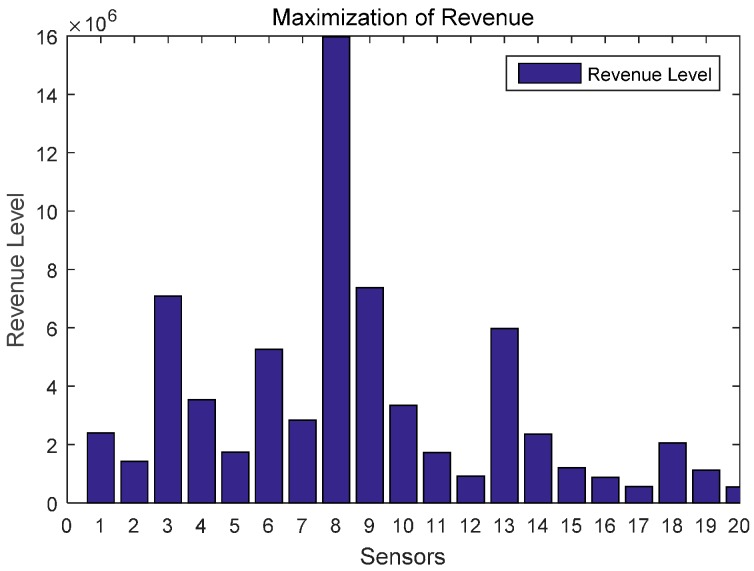
Revenue level of each sensor.

In this section, we evaluate the proposed differential game model by simulations. The simulation results of the finite-horizon and infinite-horizon differential game are both presented. We assume that the number of sensors in WPSN is N=20, and consider the time horizon T=100. Based on Equation (21), the parameter $A_{i}\left(t\right)$ of the value function $V^{i}\left(t,x\right)$ will directly impact the variation of the optimal uplink power. Thus, Figure 3 shows how the key parameter $A_{i}\left(t\right)$ varies with time. It is plotted in seconds. We observe that $A_{i}\left(t\right)$ monotonically increases for sensor 1, sensor 2, and sensor 5, monotonically decreases for sensor 3. Based on Equation (27) in Appendix A, we can see that the variation of $A_{i}\left(t\right)$ is affected by the constant parameter $\alpha_{i}$ and the energy loss coefficient $\mu_{i}$. Then, different sensors will have a different variation trend of $A_{i}\left(t\right)$. The optimal uplink transmit power of sensors under a finite-horizon are plotted in Figure 4. The optimal uplink transmit power has the same variation trend as parameter $A_{i}\left(t\right)$. In Figure 5, we show the optimal uplink transmit power under infinite-horizon. The uplink transmit power is constant and independent of time. Figure 6 explores the relationship between the optimal trajectories of the state, which are the power levels of each sensor. It can be observed that the power level has exhibits an initial growth trend. However, as the time increases, it converges to a state value. In other words, the dynamic of the power level is convergent and the convergence speed is fast. Finally, the revenue variation with time and the maximized revenue of each sensor, are evaluated and shown in Figure 7 and Figure 8. 

### 4.2. Residual Energy 

**Figure 9:**
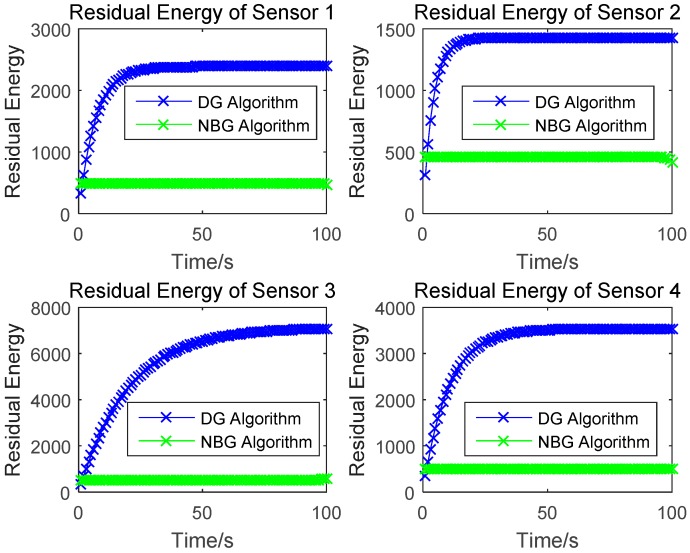
Residual energy of sensors.

In this section, we compare the proposed differential game (DG) algorithm with the Nash bargaining game (NBG) algorithm in [14], which is also a game theory-based power control method in WPSN. We use the same information transmission power for the simulations, and the test is configured with the same parameters. The residual energy of sensors one to four are shown in Figure 9. Each sensor should have a residual energy, in order to deal with information transmission tasks. As the time increases, the residual energy of the sensors under our algorithm increase, and rapidly converge to produce a stable level. The residual energy of the sensors based on the Nash bargaining game remain unchanged. Figure 9 also shows that the residual energy under our algorithm is higher than that under the NBG algorithm. Wireless sensors thus have more power for information transmission under our algorithm. 

### 4.3. QoS Revenue

**Figure 10:**
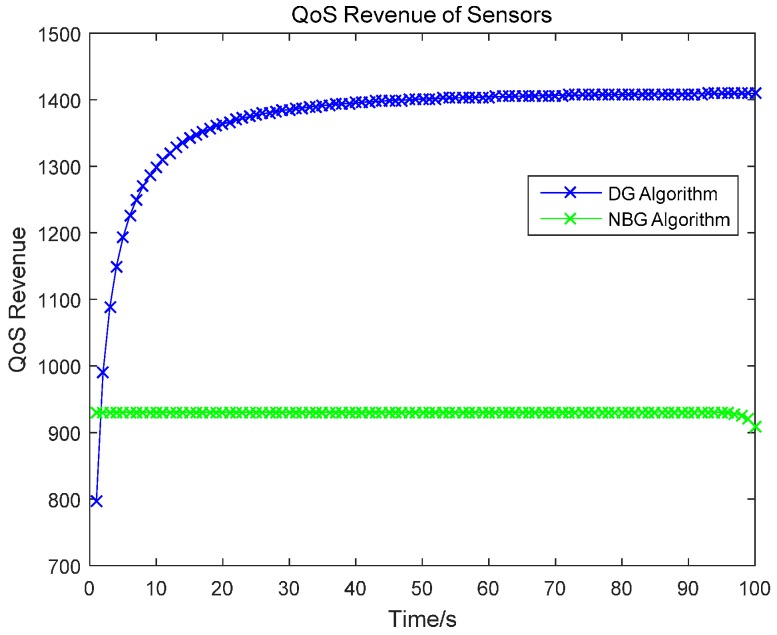
QoS revenue of sensors.

According to the QoS revenue function in Equation (4), the QoS revenue is simulated and the comparison between our DG algorithm and the NBG algorithm is shown in Figure 10. All sensors are tested in our simulations. As the time increases, because the QoS revenue is directly proportional to the energy level, the QoS revenue under the DG algorithm increases. Although the revenue under the DG algorithm is lower than that of the NBG algorithm, the increase of QoS revenue is fast. However, the QoS revenue under the NBG algorithm maintains a constant value. In addition, our algorithm reveals a better performance than the NBG algorithm.

## 5. Conclusions 

In this paper, we research the uplink transmit power control problem in wireless powered sensor networks. We propose a non-cooperative differential game model to analyze the optimal transmission power for the energy harvesting sensors. In the game, each sensor determines the uplink transmit power, to maximize the utility combination of energy revenue and QoS revenue in a time horizon. According to the Bellman dynamic programming, we can individually obtain the Nash equilibrium (NE) solutions under a finite-horizon and an infinite-horizon. When all sensors achieve NE, the optimal trajectory of the power level can be derived and the maximized revenue can be obtained. The correctness and convergence of the proposed algorithm is proved through numerical simulations. 

In future work, we will attempt to combine the power control problem and time scheduling problem, in order to analyse the buffer size influences in our model, which is more practical for the limited network resource. Then, the way in which we can achieve optimal power control under an appropriate MAC algorithm can be ascertained. Finally, the whole revenue can be maximized, based on this solution.

## Appendix A

**Proof** **of Lemma 3.** Based on the dynamic programming, let the first derivative of Equation (11) be zero, where the utility function reaches the maximum value. Thus we have:

(A1) $$p_{i}^{*}\left(s\right)=\rho V_{x}^{i}{\left(t,x\right)}^{\left(-1\right)}e^{-rt}-\frac{1}{\gamma_{i}}$$

Upon the incorporation of $p_{i}^{*}\left(s\right)$ into (11) and (12) and solving (11) and (12), we obtain the value function:

(A2) $$V^{i}\left(t,x\right)=\left[A_{i}\left(t\right)x_{i}+B_{i}\left(t\right)\right]e^{-rt}$$

where $A_{i}\left(t\right)$ and $B_{i}\left(t\right)$ satisfy:

(A3) $$dA_{i}\left(t\right)=\left[\left(r+\mu_{i}\right)A_{i}\left(t\right)-\varepsilon \right]dt$$

(A4) $$A_{i}\left(T\right)=\alpha_{i}$$

(A5) $$\begin{array}{l} dB_{i}\left(t\right)=[rB_{i}\left(t\right)-\rho \left(1-\tau_{i}\right)\log\left(1+\gamma_{i}{p_{i}}^{*}\right)\\ -A_{i}\left(t\right)\left[\eta_{i}\tau_{i}{\left\|g_{T}^{i}\right\|}^{2}p_{T}^{i}-\left(1-\tau_{i}\right)p_{i}^{*}\right]]dt \end{array}$$

(A6) $$B_{i}\left(T\right)=-\alpha_{i}S_{i}$$

Solving Equations (A3) and (A4), we have:

(A7) $$A_{i}\left(t\right)=\left(\alpha_{i}-\frac{\varepsilon }{r+\mu_{i}}\right)e^{\left(r+\mu_{i}\right)\left(t-T\right)}-\frac{\varepsilon }{r+\mu_{i}}$$

$B_{i}\left(s\right)$ can be solved as:

(A8) $$B_{i}\left(t\right)=e^{rt}\left[\int_{0}^{t}f_{i}\left(t\right)e^{-rs}ds+B_{i}^{0}\right]$$

With:

(A9) $$B_{i}^{0}=-\alpha_{i}S_{i}-\int_{0}^{T}F_{i}\left(t\right)e^{-rs}ds$$

Incorporating $A_{i}\left(t\right)$ into (21), we obtain the optimal uplink transmit power for each sensor:

(A10) $$p_{i}^{*}\left(s\right)=\rho \left[\left(\alpha_{i}-\frac{\varepsilon }{r+\mu_{i}}\right)e^{\left(r+\mu_{i}\right)\left(t-T\right)}-\frac{\varepsilon }{r+\mu_{i}}\right]-\frac{1}{\gamma_{i}}$$

Hence, Lemma 3 follows. ☐

## Appendix B

**Proof** **of Lemma 5.** Performing the indicated maximization in (16), we obtain:

(A11) $${q_{i}}^{*}=\rho W_{x}^{i}{\left(x\right)}^{\left(-1\right)}-\frac{1}{\gamma_{i}}$$

Incorporating $p_{i}^{*}\left(s\right)$ into (16) and solving (16) yields:

(A12) $$W^{i}\left(x\right)=Cx_{i}+D$$

where:

(A13) $$C=\frac{\varepsilon }{r+\mu_{i}}$$

(A14) $$D=\frac{\rho }{r}\left(1-\tau_{i}\right)\log\left(1+\gamma_{i}{q_{i}}^{*}\right)+\frac{\varepsilon }{r+\mu_{i}}\frac{1}{r}\left(\eta_{i}\tau_{i}{\left\|g_{T}^{i}\right\|}^{2}p_{T}^{i}-\left(1-\tau_{i}\right){q_{i}}^{*}\right)$$

The game equilibrium strategy can then be expressed as:

(A15) $${q_{i}}^{*}=\frac{\rho }{\varepsilon }\left(r+\mu_{i}\right)-\frac{1}{\gamma_{i}}$$

Hence, Lemma 5 follows. ☐
